# Anti-Müllerian Hormone and Cardiometabolic Disease in Women: A Two-Sample Mendelian Randomization Study

**DOI:** 10.31083/j.rcm2308269

**Published:** 2022-07-25

**Authors:** Renée M.G. Verdiesen, Joanna von Berg, M. Abdullah Said, Pim van der Harst, Anubha Mahajan, Carla H. van Gils, Yvonne T. van der Schouw, N. Charlotte Onland-Moret

**Affiliations:** ^1^Julius Center for Health Sciences and Primary Care, University Medical Center Utrecht, Utrecht University, 3584 CX Utrecht, The Netherlands; ^2^Division Laboratory, Pharmacy and Biomedical Genetics, University Medical Center Utrecht, 3584 CX Utrecht, The Netherlands; ^3^Center for Molecular Medicine, University Medical Center Utrecht, 3584 CX Utrecht, The Netherlands; ^4^Department of Cardiology, University Medical Center Groningen, University of Groningen, 9700 RB Groningen, The Netherlands; ^5^Department of Cardiology, Division Heart and Lungs, University Medical Center Utrecht, Utrecht University, 3584 CX Utrecht, The Netherlands; ^6^Wellcome Centre for Human Genetics, University of Oxford, OX3 7BN Oxford, UK

**Keywords:** anti-Müllerian hormone, AMH, coronary artery disease, stroke, type 2 diabetes, Mendelian randomization, women

## Abstract

**Background:**

Higher age-specific circulating anti-Müllerian hormone (AMH) levels have been linked to a lower risk of cardiometabolic outcomes. However, whether AMH has a casual role in the etiology of these diseases is unknown. The objective of this study was therefore to explore if circulating AMH levels have a causal effect on risk of coronary artery disease (CAD), ischemic stroke and type 2 diabetes (T2D) in women, using a two-sample Mendelian randomization (MR) approach.

**Methods:**

We used four single nucleotide polymorphisms (SNPs) from the most recent AMH GWAS meta-analysis as instrumental variables. Summary-level data for CAD (n = 149,752; 11,802 cases), ischemic stroke (n = 17,541; 4678 cases) and T2D (n = 464,389; 30,052 cases) were extracted from the UK Biobank, the Stroke Genetics Network, and DIAMANTE consortia, respectively. To assess the presence of potential pleiotropy we tested the association of the four AMH SNPs, both individually and combined in a weighted genetic risk score, with a range of cardiovascular risk factors and intermediate traits using UK Biobank data.

**Results:**

MR estimates, i.e., inverse variance-weighted odds ratios (${\mathrm{OR}}_{\text{IVW}}$), did not support a causal effect of circulating AMH levels on CAD (${\mathrm{OR}}_{\text{IVW}}$ = 1.13, 95% CI: 0.95–1.35), ischemic stroke (${\mathrm{OR}}_{\text{IVW}}$ = 1.11, 95% CI: 0.83–1.49), and T2D (${\mathrm{OR}}_{\text{IVW}}$ = 0.98, 95% CI: 0.87–1.10). After adjustment for multiple testing, we observed associations between genetically predicted AMH and age at menopause, and age at menarche, but not with intermediate traits on the causal pathway between AMH and cardiometabolic health, such as atherosclerosis or glucose levels.

**Conclusions:**

This study does not provide evidence for a causal effect of circulating AMH levels on CAD, ischemic stroke and T2D in women, although weak instrument bias cannot be excluded.

## 1. Introduction

In women, anti-Müllerian hormone (AMH) is expressed by early antral stage ovarian follicles [1]. AMH levels decline with age, and circulating levels become undetectable after menopause, when the ovarian reserve is depleted. Consequently, AMH can be used as a marker for reproductive aging [2]. Accelerated female reproductive aging, often quantified as an earlier age at menopause, has been linked to a higher risk of cardiometabolic diseases [3, 4, 5], but the causal mechanisms underlying these associations remain to be established. Based on recent observational studies that provided evidence for an association between higher circulating AMH levels and lower risk of cardiovascular disease [6], and diabetes [7], in women, it has been postulated that AMH may have a causal role in the etiology of these diseases. However, a potential causal effect of AMH on risk of cardiometabolic disease is difficult to establish in observational studies. In Mendelian randomization (MR) studies, genetic variants are used as instrumental variables for the risk factor of interest, to estimate causal effects on outcomes that are not influenced by confounding, and are not altered by disease occurrence (reverse causation) [8]. In two-sample MR, summary-level data from independent genome-wide association studies (GWASs) for the exposure and outcome(s) are used instead of individual-level data from one study population. Consequently, two-sample MR studies generally include data on a larger number of participants, which increases statistical power to detect a causal association [9].

For AMH, we have recently identified four genetic variants in ~7000 premenopausal women [10]. Using these genome-wide significant genetic variants for AMH levels, we aimed to explore if circulating AMH levels could have a causal effect on risk of cardiometabolic disease in women. Specifically, we estimated causal effects of AMH on coronary artery disease (CAD), ischemic stroke and type 2 diabetes (T2D).

## 2. Materials and Methods

### 2.1 Instrumental Variable Selection

Recently, we have identified four single nucleotide polymorphisms (SNPs) in an AMH GWAS meta-analysis that included data of 7049 premenopausal women of European ancestry [10]. One of these variants is a missense variant located in the *AMH* gene (rs10417628). However, for this SNP the possibility that it is associated with AMH levels through impaired detection by specific AMH assays, instead of reduced AMH bioactivity, could not be excluded [10, 11]. Therefore, and because inclusion of multiple genetic instruments increases statistical power to detect a causal association [12], we included all four SNPs associated with circulating AMH levels in premenopausal women at genome-wide significance (*p *< 5 × ${\mathrm{10}}^{-8}$). Details of the included genetic variants are presented in **Supplementary Table 1**. Combined, the four SNPs explained 1.47% of the variance in circulating AMH levels (i.e., $R^{2}$ = 0.0147). In all GWAS studies contributing to the AMH GWAS meta-analysis, AMH levels (pmol/L) were transformed using rank-based inverse normal transformation. As a result, presented odds ratios (ORs) for outcomes correspond to one unit increase in inverse normally transformed circulating AMH levels. In addition, each GWAS included in the AMH GWAS meta-analysis adjusted analyses for confounding due to the potential presence of distinct subpopulations in the overall study population, i.e., population stratification, (either by inclusion of the first 10 principal components or a genetic relationship matrix) and age at AMH measurement.

### 2.2 Outcome Data Sources

We included summary-level data for genetic associations of the four AMH variants with CAD, ischemic stroke and T2D in women of European descent from the UK Biobank [13], the Stroke Genetics Network (SiGN) [14, 15] and DIAMANTE [16] consortia, respectively.

The UK Biobank is a large, population-based cohort established to study the interrelationships between environment, lifestyle, and genes. The UK Biobank (https://www.ukbiobank.ac.uk) recruited over 500,000 men and women between 2006 and 2010 [13], aged 37 to 73 years at baseline. The UK Biobank was approved by the North West Multi-Centre Research Ethics Committee, and all participants provided written informed consent to participate in the study. Prevalence of CAD was determined using self-reported data as per prior analysis [17]. Additionally, the Hospital Episode Statistics “Spell and Episode” category with hospital in-patient stay diagnoses was used. CAD was defined using the International classification of disease (ICD) version 9 codes 410, 412 and 414, ICD version 10 codes I21-I25, Z951 and Z955, and the Office of Population Censuses and Surveys Classification of Interventions and Procedures, version 4 (OPCS-4) codes K40-K46, K49, K50 and K75. Controls were excluded if their father, mother, or sibling was reported to suffer from any heart disease in order to reduce biological misclassification. CAD GWAS analyses were performed using linear mixed models implemented in BOLT-LMM software [18] (v2.3.1), and adjusted for age at inclusion, genotyping array (UK Biobank Axiom or UK BiLEVE Axiom), and the first 30 principal components provided by the UK Biobank. BOLT-LMM effect estimates and standard errors were transformed to log odds ratios and corresponding standard errors as previously described [19].

The SiGN consortium is a previously compiled dataset consisting of 14,549 ischemic stroke cases of several cohorts and publicly available controls [15]. The SiGN study population has been described previously, together with details on genetic quality control and genotype imputation methodology [14]. Different procedures were used to establish ischemic stroke diagnosis, which have been described into detail elsewhere [14]. Female sex was defined as the presence of XX chromosomes. GWAS analyses for ischemic stroke were performed using BOLT-LMM [18] (v2.3.1), and adjusted for population stratification, by inclusion of a genetic relation matrix, and age. BOLT-LMM estimates for ischemic stroke were also transformed to log odds ratios and corresponding standard errors using a previously published approximation [19].

The DIAMANTE consortium included 74,124 T2D cases and 824,006 controls from 32 GWASs and has been described into detail elsewhere [16]. Studies included in DIAMANTE based T2D diagnosis on different criteria, including but not limited to, fasting glucose and HbA1c levels, hospital discharge diagnosis, use of diabetes medication, and self-report. For the current study, we requested results from sex-specific GWAS analyses, which were adjusted for population stratification and study-specific covariates [16].

There was no overlap in participants between the UK Biobank and the AMH GWAS meta-analysis. However, there may be some overlap in participants between SiGN and DIAMANTE and the AMH GWAS meta-analysis, since all three studies included participants from the Nurses’ Health Study (maximum overlap n = 642). An additional 127 participants of EPIC-Interact [20] may overlap between the AMH GWAS meta-analysis and DIAMANTE (total maximum overlap n = 769). Due to the nature of both data from the Nurses’ Health Study and EPIC-Interact study included in SiGN and DIAMANTE meta-analyses, i.e., GWAS summary-level data, we were not able to identify potential overlapping individuals. As a result, overlapping participants were not excluded.

All individual studies that were included in the GWAS meta-analyses for AMH, stroke and diabetes, and the UKBiobank cohort, received ethical approval from qualified institutional boards and all included study participants provided informed consent.

### 2.3 Statistical Analyses

We calculated MR estimates for the individual SNPs in relation to each disease outcome using the Wald ratio method. Individual Wald ratio estimates were meta-analyzed using a random-effects inverse-variance weighted (IVW) method. To assess the strength of included genetic variants for AMH we calculated F-statistics corresponding to the IVW analyses, using the proportion of variance in AMH explained by the genetic variants, the sample size of the outcome GWASs, and the number of variants included [21]. We compared overall MR estimates (i.e., IVW estimates) to SNP-specific MR estimates (i.e., Wald ratio estimates) since inconsistent estimates are indicative of horizontal pleiotropy. In addition, we tested for heterogeneity in causal effects amongst the individual SNPs using Cochrane’s Q statistics, and performed leave-one-out sensitivity analyses to assess the influence of outlying variants. For stroke, we examined whether causal associations were affected by exclusion of early onset cases (age <50 years at diagnosis), because early onset stroke is suggested to have a different etiology than stroke at older ages [22]. All MR analyses were performed using the “TwoSampleMR” package (Bristol, United Kingdom; version 0.4.25) [23] in R (Vienna, Austria; version 3.5.1) [24].

To assess potential pleiotropy (i.e., whether genetic variants are associated with multiple traits) we tested if the four AMH SNPs, either individually or combined as a genetic risk score, were associated with a range of traits in the UK Biobank. For this analysis, we selected 44 traits that were either likely to be confounders or that could affect cardiometabolic health through pathways not involving AMH (i.e., horizontal pleiotropy; e.g., active smoking and body mass index), or traits that could be intermediates in the causal pathway between AMH and cardiometabolic disease (i.e., vertical pleiotropy; e.g., markers for subclinical atherosclerosis and glycemic traits). An overview of the 44 investigated traits has been included in **Supplementary Table 2**. Depending on the type of trait linear or logistic regression models were fitted. We created a heatmap of z-scores aligned with higher genetically predicted AMH levels to visually represent potential pleiotropy. To correct for multiple testing, we considered false discovery rate (FDR) values <0.05 to be statistically significant.

## 3. Results

### 3.1 Descriptive Data Outcome Data Sources

The included number of cases and controls for each outcome are presented in Table 1 (Ref. [13, 14, 15, 16]).

**Table 1.** **Number of cases and controls for each outcome data source**.

Outcome	Study [Ref]	Number of cases	Number of controls	Age	Ancestry
Coronary artery disease	UK Biobank [13]	11,802	137,950	Cases: 62.0 (6.3)*	93.2% White British
				Controls: 55.9 (8.3)*	
Ischemic stroke	SiGN [14, 15]	4678	12,863	Cases ≥50 years: 77**	European
Age at onset ≥50 years		4247	12,863	Cases <50 years: 42**	
Type 2 diabetes	DIAMANTE [16]	30,053***	434,336***	Unavailable	European

Abbreviations: SiGN, Stroke Genetics Network. *Mean (sd). **Median. ***These numbers were extracted from the DIAMANTE GWAS meta-analysis manuscript [16]; the actual number of female cases and controls for whom data on the four AMH SNPs was available was not provided.

### 3.2 CAD

We did not find evidence for a causal association between circulating AMH levels and CAD risk (${\mathrm{OR}}_{\text{IVW}}$ = 1.13, 95% CI: 0.95–1.35) (Table 2). Results from single SNP analyses for the variants in the *AMH*, *CDCA7* and *MCM8* loci also did not support a causal association with CAD (Table 2), but we observed a risk increasing effect of the SNP in the *TEX41* locus (OR = 1.43, 95% CI: 1.07–1.91). The heterogeneity test for the IVW estimate did not indicate heterogeneous effects of the individual SNPs (Cochran’s Q = 4.42, *p* = 0.22). Leave-one-out sensitivity analyses showed that exclusion of the SNP in the *CDCA7* locus resulted in a significant association between genetically predicted circulating AMH levels and CAD risk, although the IVW effect estimate did not change considerably (${\mathrm{OR}}_{\text{IVW}}$ = 1.19, 95% CI: 1.00–1.42; **Supplementary Fig. 1**).

**Table 2.** **Mendelian randomization estimates for causal effects of circulating AMH levels on coronary artery disease, ischemic stroke and type 2 diabetes in women**.

Outcome	Method	F-statistic	Odds Ratio	95% CI	*p*-value
Coronary artery disease	IVW	558.5	1.13	0.95–1.35	0.18
	Wald ratio estimate for rs10417628 (*AMH*)		1.06	0.82–1.37	0.65
	Wald ratio estimate for rs13009019 (*TEX41*)		1.43	1.07–1.91	0.02
	Wald ratio estimate for rs16991615 (*MCM8*)		1.15	0.85–1.57	0.37
	Wald ratio estimate for rs11683493 (*CDCA7*)		0.92	0.67–1.26	0.60
Ischemic stroke	IVW	65.4	1.11	(0.83–1.49)	0.48
	Wald ratio estimate for rs10417628 (*AMH*)		1.31	(0.78–2.20)	0.30
	Wald ratio estimate for rs13009019 (*TEX41*)		0.97	(0.55–1.70)	0.90
	Wald ratio estimate for rs16991615 (*MCM8*)		0.85	(0.46–1.59)	0.62
	Wald ratio estimate for rs11683493 (*CDCA7*)		1.35	(0.71–2.56)	0.35
Type 2 diabetes	IVW	1732.1	0.98	(0.87–1.10)	0.74
	Wald ratio estimate for rs10417628 (*AMH*)		1.01	(0.83–1.23)	0.93
	Wald ratio estimate for rs13009019 (*TEX41*)		0.91	(0.72–1.15)	0.43
	Wald ratio estimate for rs16991615 (*MCM8*)		0.99	(0.77–1.26)	0.93
	Wald ratio estimate for rs11683493 (*CDCA7*)		1.01	(0.79–1.30)	0.93

Odds ratio and 95% CI are per 1 unit increase in inverse normally transformed AMH. AMH, anti-Müllerian hormone; IVW, inverse variance weighted.

### 3.3 Ischemic Stroke

The IVW estimate did not provide clear evidence for a causal association between higher genetically predicted AMH levels and risk of ischemic stroke (${\mathrm{OR}}_{\text{IVW}}$ = 1.11, 95% CI: 0.83–1.49). Wald ratio estimates for the individual genetic variants did also not support a causal association with ischemic stroke (Table 2). Causal effects across the four genetic variants were not heterogeneous (Cochran’s Q = 1.69, *p* = 0.64). Leave-one-out analyses suggested that IVW results would not change after exclusion of any of the SNPs (**Supplementary Fig. 1**).

Exclusion of women younger than 50 years of age at stroke diagnosis attenuated IVW estimates (${\mathrm{OR}}_{\text{IVW}}$ = 0.95, 95% CI: 0.70–1.27) and effect estimates for the SNPs in the *AMH*, *CDCA7* and *TEX41* loci (**Supplementary Table 3**). The effect estimate for the *MCM8* locus changed to a risk increasing effect on ischemic stroke in women aged older than 50 at diagnosis, but its confidence interval was very wide and still included the null (OR = 1.14, 95% CI: 0.60–2.17).

### 3.4 T2D

IVW MR estimates did not support an association between genetically predicted AMH and T2D (${\mathrm{OR}}_{\text{IVW}}$ = 0.98, 95% CI: 0.87–1.10). Results from the single SNP analyses also did not indicate causal associations with T2D risk (Table 2). The heterogeneity test statistic did not suggest heterogeneous effects amongst the four SNPs (Cochran’s Q = 0.54, *p* = 0.91), and leave-one-out analyses indicated that none of the SNPs had outlying effects (**Supplementary Fig. 1**).

### 3.5 Associations between Genetic Instruments for AMH and Possible Pleiotropic Traits

**Fig. 1.**
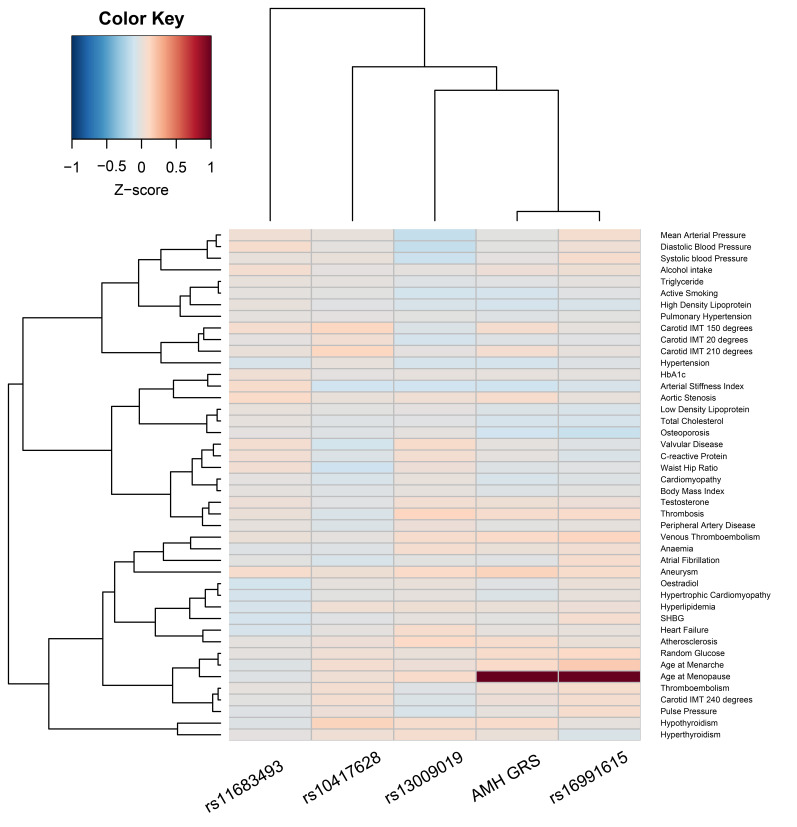
**Heatmap of associations between the individual genetic variants for AMH and the weighted genetic risk score (AMH GRS) and 44 traits of the UK Biobank**. The heatmap presents z-scores for 44 UK Biobank traits that correspond to higher genetically predicted AMH levels. Only associations between rs16991615 (*MCM8* locus) and age at menopause and age at menarche, and the association between the AMH GRS and age at menopause were statistically significant at false discovery rate <0.05. Abbreviations: IMT, intima-media thickness; SHBG, sex hormone binding globulin.

Associations between the individual AMH SNPs, and the weighted genetic risk score including all four variants, and possible pleiotropic traits are presented in Fig. 1. After correction for multiple testing, we observed a positive significant association between the SNP in the *MCM8* locus (rs16991615) and age at menopause and age at menarche. The weighted genetic risk score was only associated with age at menopause. We did not find associations with intermediate traits on the causal pathway between AMH and cardiometabolic health, such as subclinical atherosclerosis or HbA1c and glucose levels.

## 4. Discussion

Our MR analyses did not provide evidence for causal effects of circulating AMH levels on the development of CAD, ischemic stroke and T2D in women. However, due to the limited number of genetic instruments, these findings should be interpreted with due caution.

Genetic instruments used for MR analyses have to meet the following assumptions to yield valid MR estimates: (1) genetic variants have to be strongly associated with the exposure; (2) genetic variants cannot be associated with confounders of the studied associations; and (3) genetic variants cannot affect the studied outcomes through mechanisms that do not involve the exposure [25]. To meet the first criterion we only included SNPs associated with circulating AMH levels at genome-wide significance as genetic instruments. We also quantified the strength of the combination of these four SNPs through calculation of F-statistics for each outcome (558.5 for CAD, 65.4 for ischemic stroke, and 1732.1 for T2D). Although a F-statistic higher than 10 is considered to indicate a strong genetic instrument, the estimated F-statistics may be overestimated due to the use of the $R^{2}$ from the discovery AMH GWAS meta-analysis. It is therefore still possible that weak instrument bias has biased our MR estimates towards the null and reduced statistical power to detect a causal effect [26]. Due to the limited number of genetic variants we were not able to assess violation of the second and the third MR assumption using robust MR methods such as MR-Egger and MR-PRESSO.

We did assess potential pleiotropy of the genetic instruments for AMH with 44 traits in the UK Biobank. These analyses did not provide evidence for associations of the genetic variants, either individually or combined into a genetic risk score, with intermediate traits on the causal pathway between AMH and cardiometabolic health, such as subclinical atherosclerosis or HbA1c and glucose levels. We also did not observe associations between genetically predicted AMH and potential confounders like body mass index and active smoking. Heterogeneity tests and leave-one-out analyses did not support bias due to horizontal pleiotropy, although their results should also be interpreted with caution due to the limited number of SNPs. Our results suggested that higher genetically predicted AMH levels are associated with age and menarche and age at menopause. Indeed, previous GWASs identified rs16991615 at the *MCM8* locus as genetic variant for age at menopause [27, 28]. Whether these associations reflect horizontal or vertical pleiotropy remains difficult to disentangle since AMH, age at menarche and age at menopause are all linked to the functional ovarian reserve [27, 29, 30].

Potential overlap in study participants between the exposure and outcome GWASs from which summary-level data were used, could bias MR estimates towards observational associations [31]. For both SiGN and DIAMANTE, numbers of overlapping participants were small compared to the total numbers in the study (642 vs 17,541 and 769 vs 464,389, respectively). We assessed the magnitude of potential bias due to sample overlap in the current study using a web application developed by Burgess *et al*. [31] (https://sb452.shinyapps.io/overlap) , and observed that, if anything, this bias would have been minimal for both ischemic stroke and T2D. Moreover, MR estimates for each outcome indicated null effects, whereas previous observational studies showed that higher AMH levels were associated with risk of cardiometabolic disease [6, 7]. Therefore, the effect of this type of bias on the MR estimates seems negligible.

We are aware of one previous MR study on AMH, looking at the association with ischemic heart disease in men and women [32], using genetic variants that were significant in male adolescents only [33]. In contrast with our results, this MR provided some evidence for an association of higher genetically predicted AMH levels with a lower risk of ischemic heart disease in women and men combined, yet the validity of this finding is questionable since the used genetic instruments violated the first MR assumption of being strongly related to AMH levels in females. In addition, no details about possible heterogeneous effects across the individual SNPs were described.

Our findings are not in agreement with observational studies that found that women with higher age-specific AMH levels had a lower risk of these cardiometabolic diseases [6, 7]. On the other hand, previous MR studies investigating the causal effect of age at menopause, another indicator for reproductive aging, on CAD also did not find evidence for a causal association [34, 35]. To date, no MR studies investigated whether age at menopause may be causally associated with stroke or diabetes.

An explanation for the discrepancy between the observational and MR findings for the relation between AMH, but also other indicators of reproductive aging, and cardiometabolic disease may be residual confounding by (biological) aging. Given its role in ovarian follicle development and the expression of AMH in these follicles, lower AMH levels are strongly correlated with higher age in women. Also, decelerated reproductive aging, corresponding to higher age-specific AMH levels, has been linked to longevity [36, 37]. Future studies in which both circulating AMH levels and markers for biological aging (e.g., DNA methylation) are available could explore this hypothesis.

Another explanation for the discrepancy with observational findings may be that signaling factors that are either upstream or downstream of AMH in the same pathway, instead of AMH itself, are causally associated with risk of cardiovascular disease. Among the suggested upstream regulators of AMH is BMP4 [38], and reported downstream targets of AMH include NF-κB [39, 40, 41], which have both been linked to cardiovascular disease [42, 43]. Specifically, both AMH and bone morphogenetic proteins (BMPs) are members of the TGF-β superfamily of regulatory polypeptides [44], which have been identified as important regulators of atherosclerosis [45]. Expression of the AMH-specific receptor (AMHR2) in aortic and heart tissue in both mice [46] and humans [47, 48], suggests that AMH signaling indeed has a function in the vascular system, potentially through the activation of NF-κB [49]. Hence, BMP and/or NF-κB signaling may be the common underlying pathophysiological pathway explaining the observational association between AMH and cardiometabolic disease. A mediation analysis including separate genetic instruments for AMH, BMP4 and NF-kB could be used to address this knowledge gap [50].

## 5. Conclusions

In conclusion, our results do not support a causal effect of circulating AMH levels on CAD, ischemic stroke and T2D in women. These results should be interpreted carefully, since bias towards the null due to weak instrument bias in our analyses cannot be excluded.

## Supplementary Material

Supplementary material associated with this article can be found, in the online version, at https://doi.org/10.31083/j.rcm2308269.
