# Supplementary material for: Anti-Müllerian Hormone and Cardiometabolic Disease in Women: A Two-Sample Mendelian Randomization Study
Source: Rev Cardiovasc Med. 2022 Jul 25;23(8):269. doi: 10.31083/j.rcm2308269 (PMC11266950; doi:10.31083/j.rcm2308269)
Supplement: Supplementary file 1 [file 2153-8174-23-8-269-s1.zip › Supplemental Figure 1_RiCM.pdf]

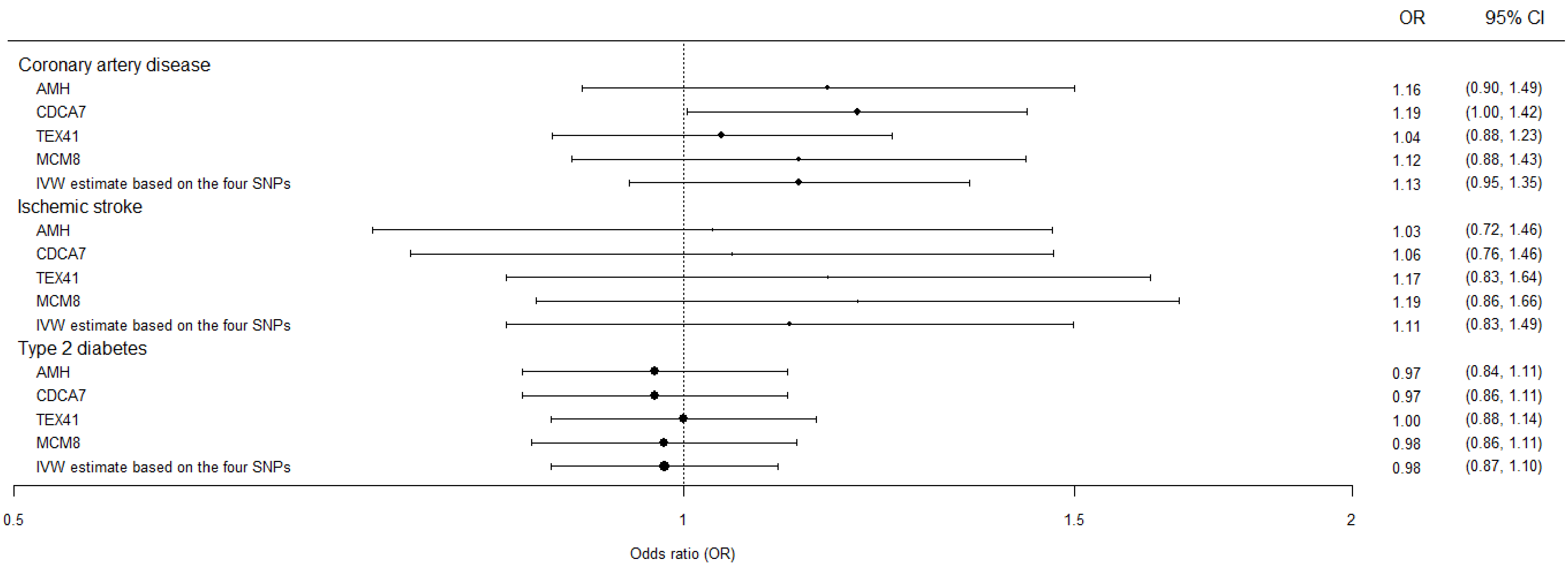

Supplemental Figure 1: Estimates of leave-one-out analyses for the association between circulating AMH and coronary artery disease, ischemic stroke and type 2 diabetes in women.
