# Supplementary material for: Anti-Müllerian Hormone and Cardiometabolic Disease in Women: A Two-Sample Mendelian Randomization Study
Source: Rev Cardiovasc Med. 2022 Jul 25;23(8):269. doi: 10.31083/j.rcm2308269 (PMC11266950; doi:10.31083/j.rcm2308269)
Supplement: Supplementary file 1 [file 2153-8174-23-8-269-s1.zip › Supplemental Table 1_MendeleyData_RiCM.docx]

Supplementary Table 1. Genetic variants associated at (*p* < 5 × 10^-8^) with inverse normally transformed AMH levels in premenopausal women.

| Locus | SNP | Chromosome | Base pair position | EA | OA | EAF | Imputation quality | Effect (SE) | *p*-value | Percentage of variance in AMH explained |
| --- | --- | --- | --- | --- | --- | --- | --- | --- | --- | --- |
| *AMH* | rs10417628 | 19 | 2251817 | T | C | 0.02 | 0.83 | –0.34 (0.05) | 1.2 × 10^-11^ | 0.50% |
| *TEX41* | rs13009019 | 2 | 145670572 | A | G | 0.69 | 0.95 | –0.09 (0.01) | 7.2 × 10^-10^ | 0.35% |
| *MCM8* | rs16991615 | 20 | 5948227 | A | G | 0.07 | 0.99 | 0.16 (0.03) | 1.2 × 10^-8^ | 0.30% |
| *CDCA7* | rs11683493 | 2 | 174259325 | T | C | 0.57 | 0.97 | –0.08 (0.01) | 1.7 × 10^-8^ | 0.32% |

Reproduced with permission from [10].

AMH, anti-Müllerian hormone; SNP, single nucleotide polymorphism; EA, effect allele; OA, other allele; EAF, effect allele frequency; SE, standard error.
