# Supplementary material for: Anti-Müllerian Hormone and Cardiometabolic Disease in Women: A Two-Sample Mendelian Randomization Study
Source: Rev Cardiovasc Med. 2022 Jul 25;23(8):269. doi: 10.31083/j.rcm2308269 (PMC11266950; doi:10.31083/j.rcm2308269)
Supplement: Supplementary file 1 [file 2153-8174-23-8-269-s1.zip › Supplemental Table 2_RiCM.docx]

| **UK Biobank trait** |
| --- |
| Pulmonary hypertension |
| Cardiomyopathy |
| Valvular disease (incl. endocarditis) |
| Aortic stenosis |
| Peripheral artery disease in extremities (incl. aneurysms) |
| Atrial fibrillation/Atrial flutter |
| Heart failure |
| Aneurysm (any location) |
| Hypertrophic cardiomyopathy |
| Thromboembolism |
| Thrombosis |
| Venous thromboembolism |
| Osteoporosis (incl medication) |
| Anaemia |
| Hypertension (incl. touchscreen medication) |
| Atherosclerosis |
| Hyperlipidemia (incl. medication) |
| Active smoker |
| Hyperthyroidism |
| Hypothyroidism |
| Diastolic blood pressure, mean across manual & automatic |
| Systolic blood pressure, mean across manual & automatic |
| Pulse pressure, mean across manual & automatic |
| Mean arterial pressure, mean across manual & automatic |
| Arterial Stiffness |
| Carotid intima-media thickness, mean at 120 degrees |
| Carotid intima-media thickness, mean at 150 degrees |
| Carotid intima-media thickness, mean at 210 degrees |
| Carotid intima-media thickness, mean at 240 degrees |
| C-reactive protein (high-sensitivity) in mg/L |
| Body mass index (kg/m^2^) |
| Waist hip ratio |
| Low density lipoprotein in mmol/L |
| High density lipoprotein in mmol/L |
| Total cholesterol in mmol/L |
| Triglyceride in mmol/L |
| HbA1c mmol/mol |
| Glucose in mmol/L |
| Log2 Interpolated Alcohol in UK Units (8 mg or 10 mL Alc) per week |
| Age at Menarche |
| Age at Menopause |
| Oestradiol in pmol/L |
| Sex hormome binding globulin in nmol/L |
| Testosterone in nmol/L |

Supplementary Table 2. Overview of the 44 UK Biobank traits tested for an association with the individual genetic variants for AMH and the weighted genetic risk score.
