# Supplementary material for: Anti-Müllerian Hormone and Cardiometabolic Disease in Women: A Two-Sample Mendelian Randomization Study
Source: Rev Cardiovasc Med. 2022 Jul 25;23(8):269. doi: 10.31083/j.rcm2308269 (PMC11266950; doi:10.31083/j.rcm2308269)
Supplement: Supplementary file 1 [file 2153-8174-23-8-269-s1.zip › Supplemental Table 3_RiCM.docx]

Supplementary Table 3. Mendelian Randomization estimates for causal effects of circulating AMH levels on ischemic stroke in women ≥50 years at diagnosis.

| **Outcome** | **Method** | **Odds Ratio** | **95% CI** | ***p*** |
| --- | --- | --- | --- | --- |
| Ischemic stroke in age onset ≥50 years | IVW | 0.95 | 0.70–1.27 | 0.72 |
|  | Wald ratio estimate for rs10417628 (*AMH*) | 0.87 | 0.52–1.46 | 0.60 |
|  | Wald ratio estimate for rs13009019 (*TEX41*) | 0.86 | 0.48–1.54 | 0.61 |
|  | Wald ratio estimate for rs16991615 (*MCM8*) | 1.14 | 0.60–2.17 | 0.70 |
|  | Wald ratio estimate for rs11683493 (*CDCA7*) | 1.01 | 0.53–1.96 | 0.97 |

AMH, anti-Müllerian hormone; IVW, inverse variance weighted.

Odds ratio and 95% CI are per 1 unit increase in inverse normally transformed AMH.
